# System Performance Corresponding to Bacterial Community Succession after a Disturbance in an Autotrophic Nitrogen Removal Bioreactor

**DOI:** 10.1128/mSystems.00398-20

**Published:** 2020-07-21

**Authors:** Hsiao-Pei Lu, Yung-Hsien Shao, Jer-Horng Wu, Chih-hao Hsieh

**Affiliations:** a Department of Biotechnology and Bioindustry Sciences, National Cheng Kung University, Tainan, Taiwan; b Department of Environmental Engineering, National Cheng Kung University, Tainan, Taiwan; c Institute of Oceanography, National Taiwan University, Taipei, Taiwan; d Research Center for Environmental Changes, Academia Sinica, Taipei, Taiwan; e Institute of Ecology and Evolutionary Biology, Department of Life Science, National Taiwan University, Taipei, Taiwan; f National Center for Theoretical Sciences, Taipei, Taiwan; United States Naval Research Laboratory

**Keywords:** community dynamics, disturbance, nitrogen removal, succession, time series, wastewater treatment

## Abstract

Performance of a bioreactor is affected by complex microbial consortia that regulate system functional processes. Studies so far, however, have mainly emphasized the selective pressures imposed by operational conditions (i.e., deterministic external physicochemical variables) on the microbial community as well as system performance, but have overlooked direct effects of the microbial community on system functioning. Here, using a bioreactor with ammonium as the sole substrate under controlled operational settings as a model system, we investigated succession of the bacterial community after a disturbance and its impact on nitrification and anammox (anaerobic ammonium oxidation) processes with fine-resolution time series data. System performance was quantified as the ratio of the fed ammonium converted to anammox-derived nitrogen gas (N_2_) versus nitrification-derived nitrate (npNO_3_^−^). After the disturbance, the N_2_/npNO_3_^−^ ratio first decreased, then recovered, and finally stabilized until the end. Importantly, the dynamics of N_2_/npNO_3_^−^ could not be fully explained by physicochemical variables of the system. In comparison, the proportion of variation that could be explained substantially increased (tripled) when the changes in bacterial composition were taken into account. Specifically, distinct bacterial taxa tended to dominate at different successional stages, and their relative abundances could explain up to 46% of the variation in nitrogen removal efficiency. These findings add baseline knowledge of microbial succession and emphasize the importance of monitoring the dynamics of microbial consortia for understanding the variability of system performance.

**IMPORTANCE** Dynamics of microbial communities are believed to be associated with system functional processes in bioreactors. However, few studies have provided quantitative evidence. The difficulty of evaluating direct microbe-system relationships arises from the fact that system performance is affected by convolved effects of microbiota and bioreactor operational parameters (i.e., deterministic external physicochemical forcing). Here, using fine-resolution time series data (daily sampling for 2 months) under controlled operational settings, we performed an in-depth analysis of system performance as a function of the microbial community in the context of bioreactor physicochemical conditions. We obtained statistically evaluated results supporting the idea that monitoring microbial community dynamics could improve the ability to predict system functioning, beyond what could be explained by operational physicochemical variables. Moreover, our results suggested that considering the succession of multiple bacterial taxa would account for more system variation than focusing on any particular taxon, highlighting the need to integrate microbial community ecology for understanding system functioning.

## Abstract (precis)

Dynamics of microbial communities are believed to be associated with system functional processes in bioreactors. However, few studies have provided quantitative evidence. The difficulty of evaluating direct microbe-system relationships arises from the fact that system performance is affected by convolved effects of microbiota and bioreactor operational parameters (i.e., deterministic external physicochemical forcing). Here, using fine-resolution time series data (daily sampling for 2 months) under controlled operational settings, we performed an in-depth analysis of system performance as a function of the microbial community in the context of bioreactor physicochemical conditions. We obtained statistically evaluated results supporting the idea that monitoring microbial community dynamics could improve the ability to predict system functioning, beyond what could be explained by operational physicochemical variables. Moreover, our results suggested that considering the succession of multiple bacterial taxa would account for more system variation than focusing on any particular taxon, highlighting the need to integrate microbial community ecology for understanding system functioning.

## INTRODUCTION

Monitoring temporal changes in community assembly (i.e., succession) is crucial for understanding the variation in system properties (1–3). For example, research on plants has shown that species diversity and system functioning generally increase during succession, with higher diversity usually begetting greater functioning of the system (4, 5). Whereas succession of plant and animal communities has been well documented (6, 7, 80), microbial succession and its impact on system functioning (8–10) are less well studied. Since microorganisms are crucial components that are responsible for numerous biochemical reactions in both natural and industrial environments (11), monitoring the dynamics of microbial communities is required for understanding the functional performance of various systems (12).

System functional performance is believed to be closely associated with changes in microbial communities (13). Previous studies on the nitrogen removal in wastewater treatment systems have investigated the microbial contributions to system functioning from an engineering point of view, through identifying the involved microorganisms that express specific metabolic activities, such as ammonium-oxidizing bacteria (AOB), nitrite-oxidizing bacteria (NOB), complete ammonium-oxidizing (comammox) bacteria (14), and anaerobic ammonium-oxidizing (anammox) bacteria (15). However, only a few studies have investigated the assembly and temporal dynamics of microbial communities with the consideration of community-level impacts on the system performance of bioreactors (16–19). Considering microbial communities as consortia of complex species-species interactions that regulate system functional processes as a whole (20, 21), monitoring microbial community succession should help us understand the variability of system performance in bioreactors (22).

In previous studies, variability in microbial community and system functioning was attributed mainly to selective pressures of external deterministic parameters (such as temperature, substrate concentration, and hydraulic retention time) of bioreactors (16–18, 23), overlooking the direct effect of microbial community dynamics on system functioning. Thus, even though some associations between microbial community and bioreactor performance have been revealed (16, 17, 19, 24), it is still unclear whether these associations are driven by shared operational conditions, microbial influence on system functioning, or a combination of the two. Moreover, previous studies on microbial community succession in bioreactors were usually based on coarse (i.e., weekly or monthly) samplings; however, considering the complexity and short generation time of microbial populations, sampling efforts at finer temporal resolution are required to capture detailed changes in microbial communities and their impacts on system performance (8). Thus, in this study, we aimed to evaluate the predictive power of microbial community for explaining the variation in system functional processes, using highly resolved daily samples to quantify microbe-system relationships. Specifically, we addressed the question of whether the dynamics in microbial communities could explain the variation in system performance of the bioreactor, beyond what could be predicted by operational physicochemical conditions.

Analogous to plant succession (3), the succession of microbial communities in bioreactors could be categorized into primary and secondary successions. Since bioreactors are typically inoculated with microbial consortia from various sources (such as sludge, soil, or compost), primary succession has been reported to vary depending on inoculum source and initial community structure (25–28). Then, after a period of operation in a controlled setting, the bioreactor can be considered to reach a steady state when biomass concentration and system performance approach a constant (29–31). However, secondary succession of microbial communities might occur due to disturbances, such as overloading (32), adding new substrates (33), or modifying operational parameters (23). In this study, we focused on secondary succession of microbial communities within a bioreactor, after it had been considered to reach a steady state, and examined the responses of microbial community succession and system performance following an artificial disturbance which consisted of homogenizing all microbial consortia (including biofilm and suspended sludge) and resuspending the mixture in the bioreactor. Specifically, to reveal the direct association between microbial community and bioreactor performance, the system would be maintained under similar operational parameters before and after the disturbance event. However, even under similar operational settings, the microbial community might vary continually over a period of time after the disturbance; this allows us to statistically examine the community effects on system performance in the context of bioreactor operational conditions.

Here, we established a simplified model system focusing on autotrophic nitrogen removal processes in a bench-scale continuously stirred tank reactor (CSTR) fed with ammonium as the sole nitrogen source. A CSTR would provide complete mixing of microbial populations and substrates, enabling the precise control of operational conditions, as well as reliable sampling of microbial communities and nitrogenous compounds of the system. It was reasonably assumed that in this bioreactor, the ammonium input would be removed only through collaborative autotrophic reactions of nitrification and anammox with a very limited contribution by heterotrophic denitrification due to no organic carbon supply (34). In comparison with traditional nitrification-denitrification nitrogen removal processes, the combination of nitrification (i.e., to convert ammonium through nitrite to nitrate) and anammox (i.e., to convert nitrite and ammonium into nitrogen gas and water, with small amounts of nitrate as a by-product) reactions is more cost-effective (35) and has been increasingly used as a green wastewater treatment process (36), although the system stability is sensitive to oxygen supply (37). Specifically, various types of microorganisms (including AOB, NOB, comammox bacteria, and anammox bacteria) associated with the efficiency of autotrophic nitrogen removal processes can simultaneously conduct aerobic and anaerobic nitrogen conversion reactions at a low dissolved-oxygen (DO) concentration (38). Owing to potentially high variation in community membership, this system represents a suitable model for investigating the succession of microbial communities after disturbance and its impact on system performance.

In terms of system performance in an autotrophic bioreactor, previous studies usually considered either the removal efficiency of the input ammonium (39, 40) or the total nitrogen reduction between inflow and outflow (34, 41). In contrast, since we aimed to reveal the effects of the bacterial community on bioreactor nitrogen removal processes, here we defined the ratio of anammox-derived nitrogen gas (N_2_) versus nitrification-derived nitrate (npNO_3_^−^) from the fed ammonium as an index of system performance. Here, we used the N_2_/npNO_3_^−^ ratio as an index, because enhancing the conversion of ammonium to nitrogen gas without nitrate accumulation in the bioreactor is the objective of the autotrophic nitrogen removal system (41). Instead of focusing on the removal ratio of ammonium-nitrogen or total nitrogen, the N_2_/npNO_3_^−^ ratio might better represent the variation and balance of autotrophic nitrogen removal processes in the system, with the consideration of the dynamics of bacteria that are responsible for those processes.

In the present case, we centered on the postdisturbance dynamics of bacterial communities and the corresponding changes in nitrogen removal processes. To reveal direct effects of the bacterial community on system performance, we conducted daily sampling after the disturbance to generate fine-resolution time series data. We characterized bacterial communities based on next-generation sequencing of the 16S rRNA gene, which provides community profiles at a high taxonomic resolution. Two major questions were addressed: (i) which bacterial taxa are present in the bioreactor and how their relative abundances change over time, and (ii) how bacterial community succession influences system performance, in the context of physicochemical conditions in the bioreactor.

## RESULTS

### Time series dynamics of bacterial communities.

**FIG 1:**
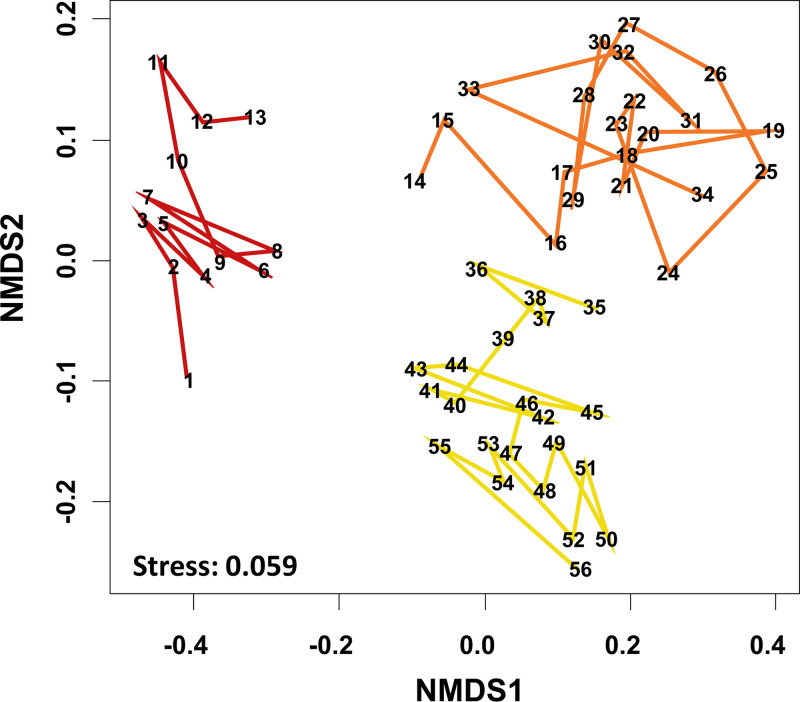
Nonmetric multidimensional scaling (NMDS) ordination, showing the time series trajectory (from 1 to 56) of bacterial composition. Time points are divided into three successional stages, indicated by different colors, based on the results of hierarchical clustering (Fig. S2).

After the disturbance, the bacterial composition changed gradually through time, with communities sampled closer together containing more similar composition profiles (Fig. S1). According to hierarchical clustering (Fig. S2), the sampled communities could be divided into three groups from time points corresponding to three successional stages (Table S1): (i) the early stage contained time points 1 to 13, (ii) the middle stage contained time points 14 to 34, and (iii) the last stage contained time points 35 to 56, with communities in the middle stage showing higher daily variation (Fig. S3). A clear succession of bacterial communities was reflected in the time series trajectory on the nonmetric multidimensional scaling (NMDS) ordination (Fig. 1).

10.1128/mSystems.00398-20.1 FIG S1 Increasing Bray–Curtis dissimilarity for pairs of communities against gaps of sampling time points. The red point shows the mean for each time gap group. Download FIG S1, PDF file, 0.1 MB. Copyright © 2020 Lu et al. 2020 Lu et al. This content is distributed under the terms of the Creative Commons Attribution 4.0 International license.

10.1128/mSystems.00398-20.2 FIG S2 Dendrogram of hierarchical clustering of bacterial communities, showing clusters of time series points corresponding to three successional stages. Download FIG S2, PDF file, 0.03 MB. Copyright © 2020 Lu et al. 2020 Lu et al. This content is distributed under the terms of the Creative Commons Attribution 4.0 International license.

10.1128/mSystems.00398-20.3 FIG S3 Box plots showing the distribution of all pairwise compositional differences of bacterial communities within each of the three successional stages. Download FIG S3, PDF file, 0.03 MB. Copyright © 2020 Lu et al. 2020 Lu et al. This content is distributed under the terms of the Creative Commons Attribution 4.0 International license.

10.1128/mSystems.00398-20.6 TABLE S1 Time series samples of bacterial communities after the disturbance based on 16S rRNA gene sequencing. Download Table S1, PDF file, 0.05 MB. Copyright © 2020 Lu et al. 2020 Lu et al. This content is distributed under the terms of the Creative Commons Attribution 4.0 International license.

**FIG 2:**
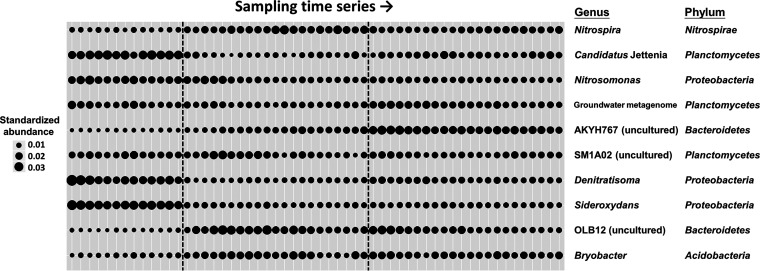
Succession of the top 10 dominant bacterial genera. Temporal changes in abundance (the aggregation of all the ASVs affiliated with each genus) have been standardized for each genus over time. The dashed lines separate data into the three successional stages, corresponding to the colors in Fig. 1.

Focusing on temporal dynamics of the top 10 dominant bacterial genera (containing >70% of the abundance in total), distinct genera varied substantially in relative abundance during succession (Fig. 2), with some being associated with earlier stages and others being associated with later stages (Table S2). Some of those genera served as indicators when their occurrences or abundances reflected the characteristics of particular successional stages (Table S2). For example, the top two dominant genera, *Nitrospira* (28.75% on average; belonging to the phylum *Nitrospirae*) and “*Candidatus* Jettenia” (21.44%; belonging to the phylum *Planctomycetes*), displayed opposite abundance trends during the three stages of succession (Fig. 2 and Table S2). In addition, *Nitrosomonas*, *Denitratisoma*, and *Sideroxydans* (all three genera belonging to the phylum *Proteobacteria*) were found to be relatively abundant in the early stage, whereas two uncultured genera (AKYH767 and OLB12) of the phylum *Bacteroidetes* tended to increase in later time points (Fig. 2 and Table S2).

10.1128/mSystems.00398-20.7 TABLE S2 Top 10 dominant bacterial genera detected in the bioreactor, showing distinct relative abundances (means ± standard deviations) in the three successional stages (*n* = 13, 21, and 22 for the early, middle, and last stage, respectively, as labeled in Table S1). Download Table S2, PDF file, 0.1 MB. Copyright © 2020 Lu et al. 2020 Lu et al. This content is distributed under the terms of the Creative Commons Attribution 4.0 International license.

Moreover, bacterial community diversity in terms of species richness, Shannon’s diversity, and Pielou’s evenness also showed temporal variation (Fig. S4). After the disturbance, species richness gradually increased over time and showed relatively high values in the final successional stage at time points 50 to 56. In contrast, Shannon’s diversity and Pielou’s evenness tended to peak around the middle stage at time points 14 to 23 (Fig. S4 and Table S3a).

10.1128/mSystems.00398-20.4 FIG S4 Fluctuations of bacterial community diversity indices (a to c) and bioreactor operational physicochemical factors during sampling (d to f). The smoothing dashed lines of the diversity indices reflect the changing trends using a moving average of 6.25 day (i.e., the hydraulic retention time). Download FIG S4, PDF file, 0.1 MB. Copyright © 2020 Lu et al. 2020 Lu et al. This content is distributed under the terms of the Creative Commons Attribution 4.0 International license.

10.1128/mSystems.00398-20.8 TABLE S3 Mean and variation of bacterial community diversity indices (a) and bioreactor operational physicochemical factors (b) in the three successional stages, as well as the whole time series. Download Table S3, PDF file, 0.1 MB. Copyright © 2020 Lu et al. 2020 Lu et al. This content is distributed under the terms of the Creative Commons Attribution 4.0 International license.

### Time series dynamics of nitrogen removal processes.

**FIG 3:**
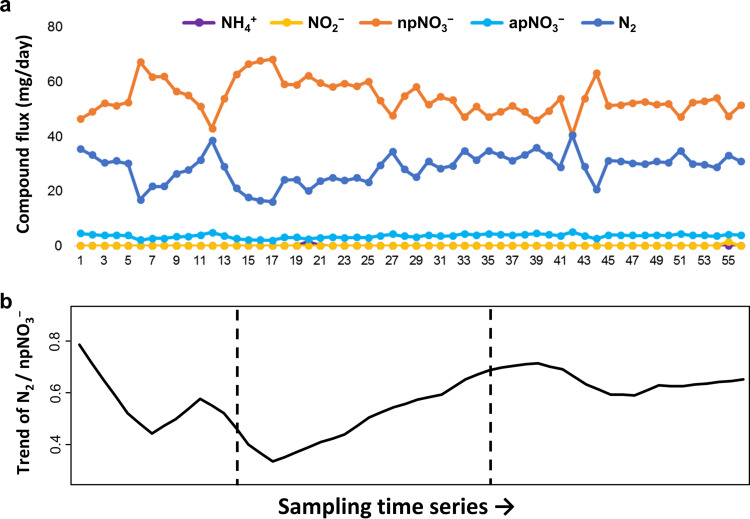
Time series of various types of nitrogen compounds (a) and a smoothing trend of N_2_/npNO_3_^−^ ratio using a moving average of 6.25 day (i.e., hydraulic retention time) (b). Here, NH_4_^+^, NO_2_^−^, and NO_3_^−^ (npNO_3_^−^ + pNO_3_^−^) were measured values, while npNO_3_^−^ (a part of NO_3_^−^ derived from the nitrification reaction), apNO_3_^−^ (a part of NO_3_^−^ derived from the anammox reaction), and N_2_ were calculated values. The dashed lines separate data into the three successional stages, corresponding to the colors in Fig. 1.

In the bioreactor, the conversion of injected ammonium (NH_4_^+^) and intermediate nitrite (NO_2_^−^) was quick and complete (below the detection limit in most sampling time points), resulting in nitrate (NO_3_^−^) and nitrogen gas (N_2_) as system end products (Fig. 3). Considering the balance of bacterium-involving biochemical reactions in this autotrophic nitrogen removal system, N_2_ was derived from anammox, while a major proportion (94% ± 2%) of NO_3_^−^ was derived from nitrification (referred to here as npNO_3_^−^) (Fig. 3a). The N_2_/npNO_3_^−^ ratio (a proxy of system performance) tended to decrease in the early stage (lowest at time points 14 to 17) but recovered in the middle stage (highest at time points 39 to 42) and then remained relatively constant in the last stage (Fig. 3b), with lower dynamics in the last stage (Table S4).

10.1128/mSystems.00398-20.9 TABLE S4 Mean, variation, and changing trends of the N_2_/npNO_3_^−^ ratio in the three successional stages based on the Mann-Kendall test and the Sen's slope. Download Table S4, PDF file, 0.1 MB. Copyright © 2020 Lu et al. 2020 Lu et al. This content is distributed under the terms of the Creative Commons Attribution 4.0 International license.

### System performance explained by different variables.

Physicochemical variables measured in this study, including temperature, pH, and DO concentration (Fig. S4 and Table S3b), could not provide high predictive power (only 9.5%) for the N_2_/npNO_3_^−^ ratio (Table 1). Rather, the proportion of variation that could be explained substantially increased when bacterial community components were incorporated into the determination (Table 1). Specifically, integrating bacterial community evenness with physicochemical variables could account for 21.8% of the explained variation. Moreover, when bacterial community structure (i.e., NMDS scores) was taken into account, 28.4% of the variation could be explained, which tripled the explained proportion compared to that obtained by considering physicochemical variables *per se*. Moreover, it is worth mentioning that the best Akaike information criterion (AIC) model suggested that compared to considering the community structure variables *per se* (accounting for 21.5% of the explained variation), the adding of alpha-diversity variables did not provide extra explained variance (Table 1), indicating the importance of focusing on the dynamics of community members. Thus, subsequently, we evaluated the effects of relative abundance changes in the top 10 dominant bacterial genera on nitrogen removal efficiency. The results revealed that each individual genus accounted for 0.5% to 9.3% of the variation in system performance, and up to 45.9% of the variation could be explained when the relative abundances of multiple bacterial genera, which were *Denitratisoma* and three uncultured candidate genera included in the best AIC model, were incorporated into the determination (Table 2).

**TABLE 1:** Results of multivariate regression for explaining the variation of N_2_/npNO_3_^−^ ratio in relation to predictor variables

Model^*a*^	Adjusted *R*^2^^*b*^	Standardized regression coefficient for:					
		Temp	DO	Evenness	NMDS1	NMDS2	NMDS3
Only ENV	**0.095**^*c*^	**0.334**^*c*^					
Only DIV	0.036			−0.231			
Only COM	**0.215**					**−0.267**	**0.416**
ENV+DIV	**0.218**	**0.329**	−0.244	**−0.371**			
ENV+COM	**0.284**	**0.349**			**−0.269**	−0.178	**0.347**
DIV+COM	**0.215**					**−0.267**	**0.416**
ENV+DIV+COM	**0.284**	**0.349**			**−0.269**	−0.178	**0.347**

a For each model, only results of the best model according to AIC scores are shown. ENV corresponds to temperature, pH, and dissolved-oxygen (DO) concentration; DIV corresponds to richness, Shannon’s diversity, and Pielou’s evenness; COM corresponds to the three main axes from the NMDS analysis.

b The adjusted *R*^2^ value (after accounting for the degree of freedom) indicates the predictive power of the best model.

c Values with significant *P* values (<0.05) are in bold.

**TABLE 2:** Results of univariate or multivariate regression models for explaining the variation of N_2_/npNO_3_^−^ ratio, in relation to the relative abundance of top 10 dominant bacterial genera

Model	Predictor variable	Coefficient	Adjusted *R*^2^
Univariate	*Nitrospira*	−0.107	0.007
	*Candidatus* Jettenia	0.166	0.009
	*Nitrosomonas*	−0.236	0.038
	Groundwater metagenome	0.233	0.037
	AKYH767 (uncultured)	**0.332**	**0.093**
	SM1A02 (uncultured)	**−0.266**	**0.054**
	*Denitratisoma*	**0.320**	**0.086**
	*Sideroxydans*	0.067	0.005
	OLB12 (uncultured)	−0.174	0.012
	*Bryobacter*	−0.081	0.007

Multivariate^*a*^	Groundwater metagenome	**−0.368**	**0.459**
	AKYH767 (uncultured)	**0.791**	
	SM1A02 (uncultured)	−0.164	
	*Denitratisoma*	**0.868**	

a For the multivariate regression model, only the best model with a subset of selected predictor variables according to AIC scores is shown. Values with significant *P* values (<0.05) are in bold.

### Functional prediction of bacterial taxa.

According to functional annotation, the removal of the fed ammonium could be attributed mainly to the predominant genera *Nitrospira*, “*Candidatus* Jettenia,” and *Nitrosomonas* (Table S5); these three genera accounted for ∼30%, ∼20%, and ∼10% of the total abundance (Table S2). *Nitrospira* could perform aerobic nitrite oxidation (as NOB) or complete oxidation of ammonium to nitrate (comammox), “*Candidatus* Jettenia” could perform anaerobic ammonium oxidation (anammox), and *Nitrosomonas* could perform aerobic ammonium oxidation (as AOB). Moreover, two uncultured genera, SM1A02 and a groundwater metagenome, were detected as potential anammox bacteria. In addition, *Denitratisoma*, as a denitrifying bacterium, together with other chemoheterotrophic bacteria might also contribute to nitrogen conversion processes in the bioreactor (Table S5).

10.1128/mSystems.00398-20.10 TABLE S5 Functional annotation of the top 10 dominant bacterial genera detected in the bioreactor. Download Table S5, PDF file, 0.1 MB. Copyright © 2020 Lu et al. 2020 Lu et al. This content is distributed under the terms of the Creative Commons Attribution 4.0 International license.

## DISCUSSION

### The bacterial community shows successional dynamics after a disturbance.

After the disturbance, the bacterial community structure varied markedly over time (Fig. 1). Specifically, individual bacterial taxa exhibited substantial variation in relative abundance over time (Fig. 2) and showed distinct associations with different successional stages (Table S2). Even though the bioreactor was operated under controlled physicochemical conditions, it is worth mentioning that the measured environmental variables of the system fluctuated after the disturbance, especially for the DO in the early stage (Fig. S4 and Table S3b). This fluctuation might be associated with the early succession of bacterial communities, similar to the phenomenon reported in other disturbance-induced community dynamics (42). In fact, ∼25% of the variation concerning bacterial community compositions could be explained by the operational physicochemical parameters (Fig. S5). However, in the middle and later stages, besides environmental variability as an external driver (43, 44), other factors, such as biotic interactions or stochastic assembly, may also play important roles in shaping bacterial community succession (44, 45). Regarding biotic interactions, previous studies have suggested that microbial consortia establish interspecies communication and specific partnerships to generate efficient metabolic processes (20). Therefore, in the middle and later successional stages, even under relatively stable physicochemical conditions, the bacterial community still varied with time; this is likely because bacterial taxa continuously interact with each other, resulting in biological internally driven community succession.

10.1128/mSystems.00398-20.5 FIG S5 Ordination biplot of distance-based redundancy analysis, showing associations between explanatory variables (temperature, dissolved-oxygen concentration, and pH) and the time series (from 1 to 56) of bacterial communities. Time points of the three successional stages are colored red, purple, and green. Download FIG S5, PDF file, 0.04 MB. Copyright © 2020 Lu et al. 2020 Lu et al. This content is distributed under the terms of the Creative Commons Attribution 4.0 International license.

### System performance varies corresponding to bacterial community succession.

The most important finding of this study is that when bacterial community components are taken into account, the explained variation for the system nitrogen removal processes substantially increases, compared to the analysis based on physicochemical variables alone (Table 1). While associations between microbial community and bioreactor performance have frequently been suggested (16, 19, 24, 29, 43), here we provide for the first time statistically evaluated results supporting the concept that microbial community succession exerts a significant influence on system functional processes of the bioreactor, beyond the effects exerted by the operational physicochemical parameters. These microbe-system relationships are expected but have hitherto been difficult to quantitatively evaluate.

In terms of the bacterial community effects, the community structure variables accounted for more explained variance than the alpha-diversity variables (Tables 1), and changes in the relative abundance of dominant bacterial genera could explain up to 46% of the variation in nitrogen removal efficiency (Tables 2). These results support the concept that monitoring the dynamics of community assembly (i.e., the presence and abundance of specific taxa in a community), rather than focusing on alpha-diversity index alone, would improve our ability to anticipate the variability of system functional processes (12). In fact, we detected a negative relationship between community evenness and system performance (Table 1), which is counterintuitive in light of typical biodiversity effects on ecosystem functioning (46). Our results suggest that the detected negative diversity-system relationship could be a consequence of the dominance effects of some species that contribute significantly to some specific functioning of the system (47, 48). That is, high dominance levels of particular functional taxa would lead to high system performance (i.e., nitrogen removal) and consequently result in the negative diversity-system relationship. That might be the case in this CSTR system.

In this system, we found that the temporal changes of taxa’s relative abundances would explain substantial variance in system performance (Table 2), reinforcing the need to account for species identity effect (47, 48). The results of regression models indicated that considering the succession of four specific bacterial taxa (including *Denitratisoma* and three uncultured genera) would explain much more system variation than focusing on any particular taxon (Table 2). Specifically, there was no significant simple correlation between either *Nitrospira* (as NOB or comammox bacteria) or “*Candidatus* Jettenia” (as anammox bacteria) and the output of nitrate or nitrogen gas. This may seem counterintuitive but, in fact, is to be expected in a working microbial system, since diverse bacterial taxa with complex metabolic interactions should be involved in the balance of nitrogen conversion processes (13). These findings suggest that dominant taxa do not function alone; they might interact closely with other community members to regulate the system processes as a whole (20, 21).

### Diverse bacterial functional groups coexist in the bioreactor.

Various types of bacterial functional groups coexisted in the bioreactor, including taxa directly involved in nitrogen conversion processes as well as a small proportion of chemoheterotrophic bacteria (Table S5). Overall, anammox and nitrification were the two major processes in this model system, as found in bioreactors with restricted aeration (36, 37).

For an autotrophic nitrogen removal bioreactor operated under conditions of low DO concentration, the anammox process is expected to combine with a partial-nitrification step (i.e., oxidizing ammonium to nitrite by AOB but not further oxidizing nitrite to nitrate by NOB) to achieve the high system performance (41). Often, supplying the oxygen at low levels is a practical measure to stimulate the growth of AOB over NOB in the partial-nitrification process (49, 50), because the oxygen half-saturation constant (*K*_O_) value of AOB is generally lower than that of NOB (51, 52). Paradoxically, our findings showed that the genus *Nitrospira* (NOB or comammox bacteria) outperformed the genus *Nitrosomonas* (AOB) (Table S2) at a low DO concentration (∼0.2 mg/liter) in the present bench-scale bioreactor, resulting in complete nitrification and accumulation of nitrate (Fig. 3). The counterexample found in this study may be explained by the recent argument that the *K*_O_ values for AOB and NOB vary greatly from one case to the other (53, 54). Some studies have reported lower *K*_O_ values for NOB than for AOB in their systems (55, 56). Thus, actual maintenance of the partial nitrification process has to be assessed case by case, particularly under different DO conditions. Further research on the *K*_O_ values of different types of AOB and NOB would help design an autotrophic nitrogen removal system which enhances the conversion of ammonium to nitrogen gas with minimal accumulation of nitrate.

Moreover, regarding the four bacterial genera with predictive power in the regression model (Table 2), two of them may function as anammox bacteria, while the other two may conduct denitrification-related and/or chemoheterotrophic reactions (Table S5). Although the heterotrophic reaction was not considered in this simplified model of the autotrophic nitrogen removal process, the collective activity of the heterotrophic bacteria may play important roles in influencing the system functioning after the disturbance. How these heterotrophic bacteria contribute to system performance and whether they interact with nitrifiers and anammox bacteria should be studied in the future.

### Conclusion.

This study paid special attention to the postdisturbance dynamics of bacterial communities and revealed the significant effects of bacterial community components on bioreactor system performance. The present study is different from previous studies in that it investigated the temporal dynamics of the bioreactor microbial community by using highly resolved daily samples to quantify microbe-system relationships. These fine-resolution time series data allowed an in-depth analysis of system performance as a function of the microbial community in the context of bioreactor operational conditions. The findings indicate that the temporal changes in bacterial community components could explain much of the variation in bioreactor functional processes beyond what could be predicted by operational physicochemical parameters alone, highlighting the need to monitor the dynamics of microbial consortia for understanding system performance of bioreactors. Validation of our findings by additional studies using parallel bioreactors, multiple disturbances in one bioreactor, and other types of disturbances would provide a more generalized conclusion.

## MATERIALS AND METHODS

### Continuously stirred tank reactor system.

A benchtop bioreactor (PolyGerm 500; Micro-Giant BioEngineering) containing a 4.5-liter working volume (in a 5-liter container) was initiated as a model system for autotrophic nitrogen removal under oxygen-limiting conditions (at agitation speeds of 40 rpm) on 17 November 2016. The bioreactor was inoculated with sludge (1,300 mg/liter) obtained from a membrane bioreactor for treatment of petrochemical wastewater and operated in CSTR mode at ∼30°C in the dark, with a hydraulic retention time of 6.25 days. The input substrate was prepared in accordance with the medium described previously (14), containing ammonium chloride (86.4 mg ammonium nitrogen [NH_4_^+^-N]/day) as the sole nitrogen source, sodium bicarbonate (40 mM) as the carbon source, and a pH buffer (pH ∼7.6). Oxygen was periodically supplied to the bioreactor with a filtration-sterilized airflow of 0.9 to 1.1 liters/day for maintaining low DO levels (∼0.2 mg/liter). Physicochemical parameters of the bioreactor, including temperature, pH, and DO, were monitored using on-line electrodes (Suntex Instruments).

### Artificial disturbance.

Prior to this study, the bioreactor had been operated for 8 months and displayed a high efficiency of converting input ammonium to nitrogen gas. On 10 July 2017, we created an artificial disturbance of the microbial consortia of the bioreactor by homogenizing all types of microbial consortia (including biofilms on the inner surface, stirrers, and pipelines as well as suspended sludge) and resuspending the mixture in the solution. After the disturbance, fine-resolution daily time series sampling was carried out for about 2 months (Table S1) to monitor the succession of bacterial communities and corresponding system functional processes.

### System performance monitoring.

Concentrations of nitrogenous compounds, including ammonium nitrogen (NH_4_^+^-N), nitrite nitrogen (NO_2_^−^-N), and nitrate-nitrogen (NO_3_^−^-N), were analyzed daily using Dionex ICS-1100 ion chromatographs (Thermo Fisher Scientific) with two columns: Dionex IonPac CS12A RFIC (for ammonium) and Dionex IonPac AS9-HC (for nitrite and nitrate). In terms of biological reactions, NH_4_^+^ is expected to be first oxidized to NO_2_^−^ by AOB and further oxidized to NO_3_^−^ by NOB (referred to in total as nitrification) aerobically, while a proportion of NH_4_^+^ with NO_2_^−^ could be anaerobically converted to N_2_ along with NO_3_^−^ as the by-product (about 0.26 mol of NO_3_^−^ per 1.02 mol of N_2_) through the reaction of anammox bacteria (57). Consequently, the nitrogen loss in the effluent was the expected nitrogen gas (N_2_) derived from the anammox, while NO_3_^−^ detected in the system was further divided according to origin, nitrification-derived NO_3_^−^ (npNO_3_^−^) and anammox-derived NO_3_^−^ (apNO_3_^−^), based on the stoichiometric balance (57).

Here, the N_2_/npNO_3_^−^ ratio was calculated as a proxy of system performance to indicate the efficiency of anammox versus nitrification nitrogen removal processes. Because of the very slow growth of autotrophic bacteria and the lack of organic carbon source, the nitrogen flux through biomass assimilation and heterotrophic denitrification in this system could reasonably be ignored. The Mann-Kendall trend test (58) was conducted to determine whether there was a monotonic upward or downward trend in the N_2_/npNO_3_^−^ ratio corresponding to the successional stages of bacterial communities, and the slope of the trend was determined with Sen’s slope (59), using the Kendall (60) and trend (61) packages in the R platform (62).

### Bacterial community monitoring.

For each sampling, about 50 ml of mixed liquors was withdrawn from the bioreactor using a syringe and then filtered through a 0.22-μm membrane to harvest microbial cells. Total microbial DNA was extracted using a bead-beating method with the DNeasy PowerWater kit (Qiagen), according to the manufacturer's instructions. To determine bacterial community structure, the V5-V6 region of the 16S rRNA gene was amplified using bacterial universal primers (787F and 1046R) (63) and subjected to a 2 × 300-bp paired-end sequencing on the Illumina MiSeq platform. The specific details regarding PCR amplification and sequencing preparation have been described previously (64). In this study, archaeal community structure was not considered, as our preliminary findings (based on quantitative PCR [qPCR] results; data not shown) suggested that there was a relatively low level of archaeal DNA compared to bacterial DNA in our study system.

### Processing of sequence data.

To minimize sequencing errors, low-quality sequences (<Q30) were trimmed out first with Trimmomatic 0.35 (65). Qualified reads were further processed using the DADA2 pipeline (66) for merging paired-end reads, removing chimeras, and inferring amplicon sequence variants (ASVs), which are finer-resolution analogues of traditional operational taxonomic units (OTUs) but without a fixed dissimilarity threshold (67, 68). Taxonomic groups (genus to phylum level) of ASVs were assigned using BLAST (E value = 10^−6^; identity > 0.9) against the SILVA 132 rRNA database (69). Moreover, the FAPROTAX database (70) was used for mapping prokaryotic taxa with functions reported in the literature.

### Bacterial community structure.

To reveal the dynamics of bacterial communities over sampling time points, diversity and composition metrics were calculated. To fairly compare community structure across samples, original data were subsampled 100 times to equal sequencing depth (30,000 sequences per sample) using the QIIME pipeline (71). For diversity metrics, species richness of each sample was calculated as the number of ASVs detected, and Shannon’s diversity and Pielou’s evenness were calculated to further weight the relative abundance patterns of ASVs (72). For composition metrics, Bray-Curtis dissimilarity was calculated to quantify the pairwise difference between samples. Then, nonmetric multidimensional scaling (NMDS) ordination was performed to visualize community succession over sampling time points. Moreover, hierarchical cluster analysis based on Ward’s method (minimizing the total within-cluster variance) combined with elbow method (determining the optimal number of clusters) was used to evaluate whether bacterial communities from the time-serial sampling points would be separated into different successional stages. Furthermore, indicator value analysis (73) was applied to reveal bacterial indicator taxa that showed preferences associated with a particular successional stage, using the point-biserial correlation coefficient as the association index (where −1 to +1 indicates a perfect negative to perfect positive association) (74). In addition, to estimate the influence of operational physicochemical factors (including temperature, pH, and DO concentration) on the compositional variation of bacterial communities, distance-based redundancy analysis (75) was performed. The statistical analyses described above were conducted using the vegan (76), factoextra (77), and indicspecies (74) packages in the R platform (62).

### Multivariate regression models.

To detect the relationship between bacterial community structure and bioreactor system processes, multivariate regression models were used to evaluate whether the variation in bioreactor performance (using the N_2_/npNO_3_^−^ ratio as a proxy; here, N_2_ and npNO_3_^−^ represent reaction end products from anammox and nitrification, respectively) could be explained by the bacterial community dynamics, in the context of physicochemical conditions. In the models, three types of predictor variables were considered, including physicochemical variables (temperature, pH, and DO concentration), diversity variables (richness, Shannon’s diversity, and Pielou’s evenness), and composition variables (specifically, NMDS1, NMDS2, and NMDS3 of the NMDS ordination were used to represent the overall community structure, while the relative abundances of the top 10 genera were used to represent the shift of taxonomic members). All variables were standardized to unit mean and variance prior to the analysis. Multivariate regression models were performed considering either single or various types of predictor variables, and the best model was selected according to the Akaike information criterion (AIC), using the MASS (78) and car (79) packages in the R platform (62).

### Data availability.

Sequencing data have been deposited in the NCBI Sequence Read Archive (SRA) under the accession number PRJNA543755.
