# Supplementary figures and images for: System Performance Corresponding to Bacterial Community Succession after a Disturbance in an Autotrophic Nitrogen Removal Bioreactor
Source: mSystems. 2020 Jul 21;5(4):e00398-20. doi: 10.1128/mSystems.00398-20 (PMC7566277; doi:10.1128/mSystems.00398-20)

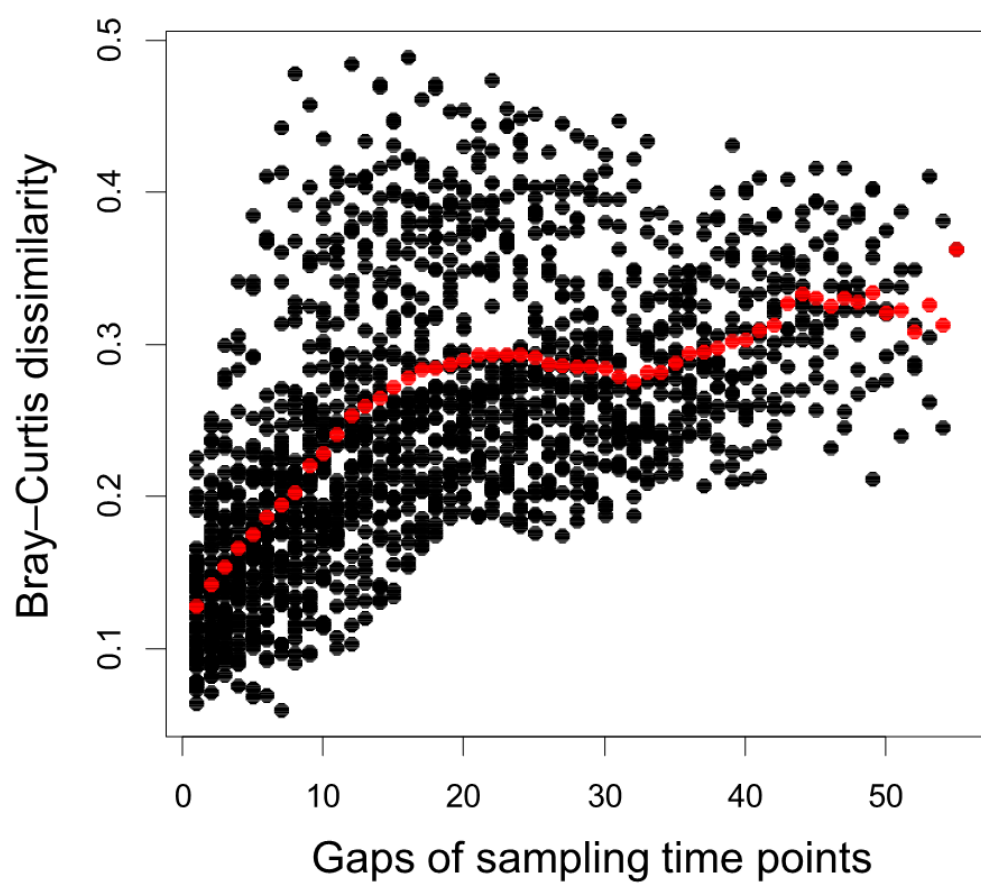

Supplement: FIG S1 [file mSystems.00398-20-sf001.pdf]

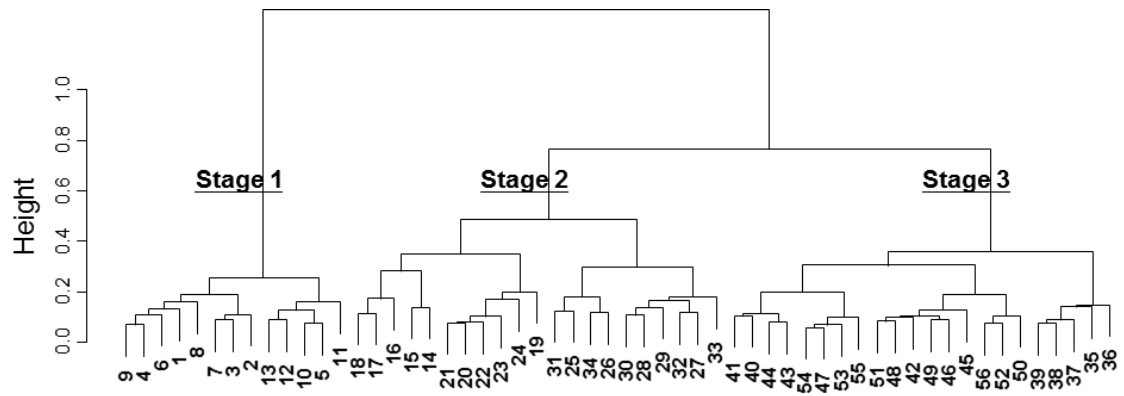

Supplement: FIG S2 [file mSystems.00398-20-sf002.pdf]

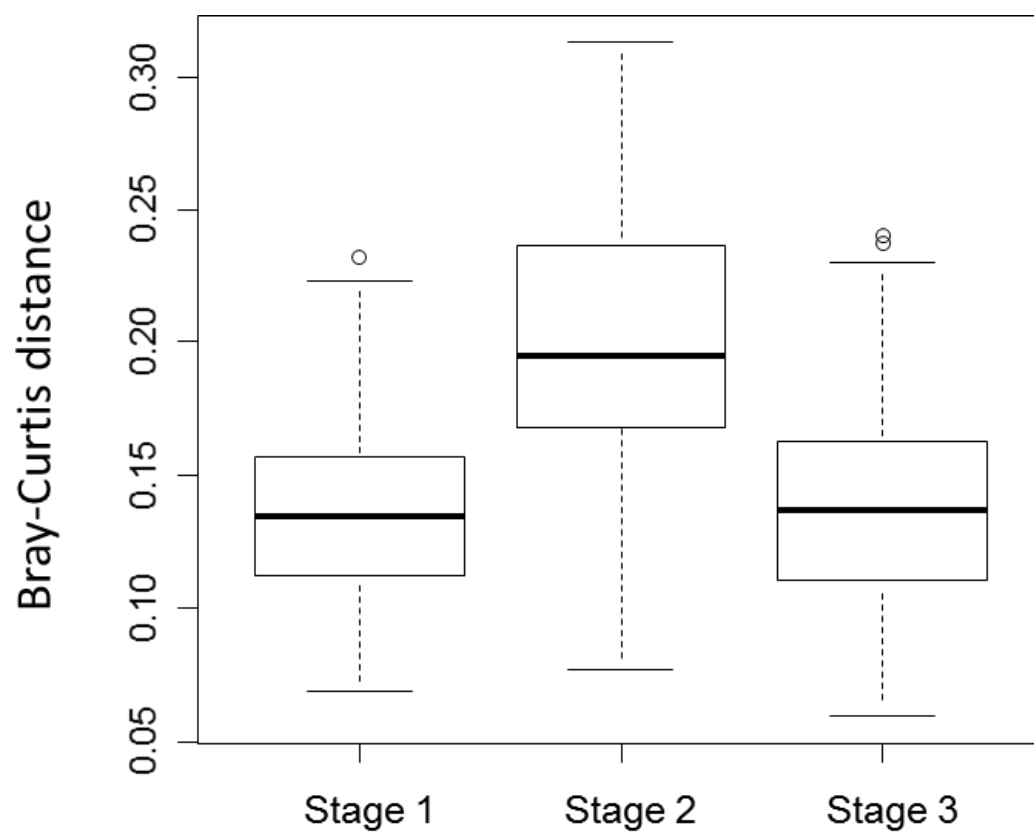

Supplement: FIG S3 [file mSystems.00398-20-sf003.pdf]

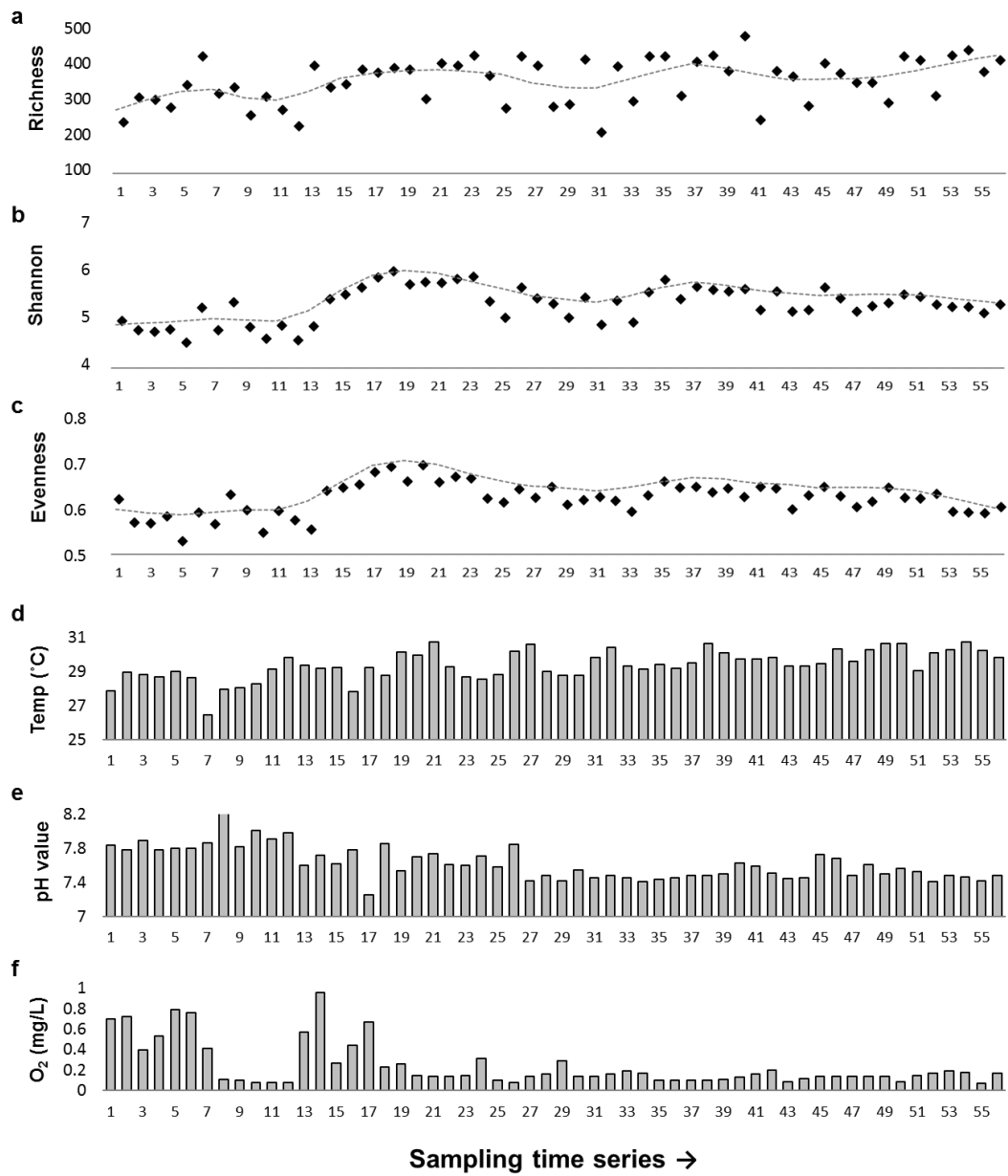

Supplement: FIG S4 [file mSystems.00398-20-sf004.pdf]

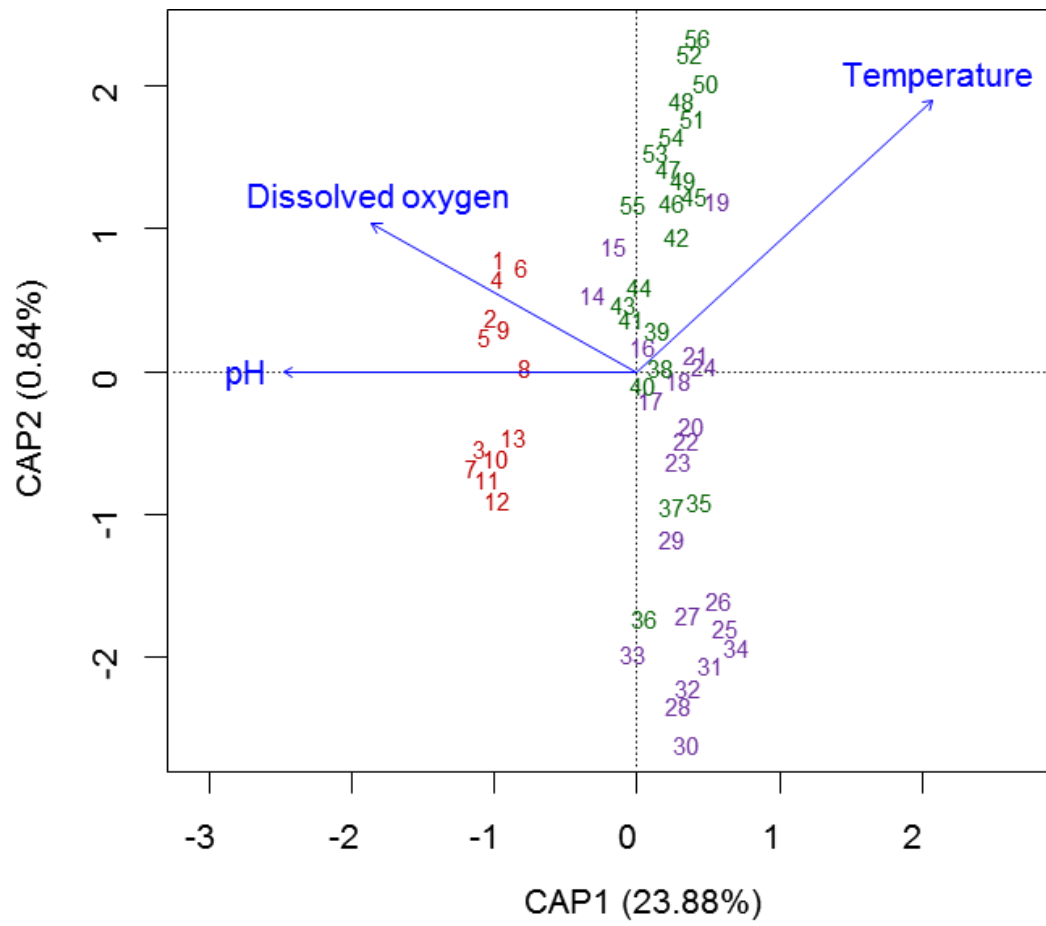

Supplement: FIG S5 [file mSystems.00398-20-sf005.pdf]
