# Supplementary material for: System Performance Corresponding to Bacterial Community Succession after a Disturbance in an Autotrophic Nitrogen Removal Bioreactor
Source: mSystems. 2020 Jul 21;5(4):e00398-20. doi: 10.1128/mSystems.00398-20 (PMC7566277; doi:10.1128/mSystems.00398-20)
Supplement: TABLE S1 [file mSystems.00398-20-st001.pdf]

Sample ID	Sampling day	Time series	Successional stage
CB0711A01	2017-07-11	1	Early
CB0712A02	2017-07-12	2	Early
CB0713A03	2017-07-13	3	Early
CB0714A04	2017-07-14	4	Early
CB0715A05	2017-07-15	5	Early
CB0716A06	2017-07-16	6	Early
CB0717A07	2017-07-17	7	Early
CB0718A08	2017-07-18	8	Early
CB0719A09	2017-07-19	9	Early
CB0720A10	2017-07-20	10	Early
CB0721A11	2017-07-21	11	Early
CB0722A12	2017-07-22	12	Early
CB0725A13	2017-07-25	13	Early
CB0728A14	2017-07-28	14	Middle
CB0731A15	2017-07-31	15	Middle
CB0803A16	2017-08-03	16	Middle
CB0806A17	2017-08-06	17	Middle
CB0808A18	2017-08-08	18	Middle
CB0811A19	2017-08-11	19	Middle
CB0812A20	2017-08-12	20	Middle
CB0813A21	2017-08-13	21	Middle
CB0814A22	2017-08-14	22	Middle
CB0815A23	2017-08-15	23	Middle
CB0817A24	2017-08-17	24	Middle
CB0818A25	2017-08-18	25	Middle
CB0819A26	2017-08-19	26	Middle
CB0820A27	2017-08-20	27	Middle
CB0823A28	2017-08-23	28	Middle
CB0824A29	2017-08-24	29	Middle
CB0825A30	2017-08-25	30	Middle
CB0826A31	2017-08-26	31	Middle
CB0827A32	2017-08-27	32	Middle
CB0828A33	2017-08-28	33	Middle
CB0829A34	2017-08-29	34	Middle
CB0830A35	2017-08-30	35	Last
CB0831A36	2017-08-31	36	Last

CB0901A37	2017-09-01	37	Last
CB0902A38	2017-09-02	38	Last
CB0903A39	2017-09-03	39	Last
CB0904A40	2017-09-04	40	Last
CB0905A41	2017-09-05	41	Last
CB0906A42	2017-09-06	42	Last
CB0907A43	2017-09-07	43	Last
CB0908A44	2017-09-08	44	Last
CB0909A45	2017-09-09	45	Last
CB0910A46	2017-09-10	46	Last
CB0911A47	2017-09-11	47	Last
CB0912A48	2017-09-12	48	Last
CB0913A49	2017-09-13	49	Last
CB0914A50	2017-09-14	50	Last
CB0915A51	2017-09-15	51	Last
CB0916A52	2017-09-16	52	Last
CB0917A53	2017-09-17	53	Last
CB0918A54	2017-09-18	54	Last
CB0919A55	2017-09-19	55	Last
CB0920A56	2017-09-20	56	Last
