# Supplementary material for: System Performance Corresponding to Bacterial Community Succession after a Disturbance in an Autotrophic Nitrogen Removal Bioreactor
Source: mSystems. 2020 Jul 21;5(4):e00398-20. doi: 10.1128/mSystems.00398-20 (PMC7566277; doi:10.1128/mSystems.00398-20)
Supplement: TABLE S2 [file mSystems.00398-20-st002.pdf]

ID	Genus	Whole	Early	Middle	Last
G1	*Nitrospira*	28.75±7.94	17.56±2.29	**34.84±5.79**#	29.55±3.85
G2	*Candidatus* Jettenia	21.44±8.44	**33.68±4.40**	14.86±4.76	20.49±4.09
G3	*Nitrosomonas*	9.16±2.16	**11.79±1.66**	8.99±1.90	7.76±0.92
G4	Groundwater metagenome	3.90±0.74	4.03±0.85	3.46±0.49	**4.23±0.68**
G5	AKYH767 (uncultured)	2.74±1.11	1.17±0.15	2.60±0.50	**3.81±0.46**
G6	SM1A02 (uncultured)	1.66±0.35	1.73±0.28	1.72±0.47	1.57±0.23
G7	*Denitratisoma*	1.50±0.57	**2.34±0.54**	1.07±0.15	1.40±0.21
G8	*Sideroxydans*	1.44±0.54	**2.36±0.29**	1.16±0.12	1.16±0.13
G9	OLB12 (uncultured)	1.03±0.44	0.44±0.08	**1.34±0.31**	1.09±0.30
G10	*Bryobacter*	0.94±0.27	0.58±0.13	**1.06±0.24**	1.03±0.16

#The value in bold indicates a significant association (p-value < 0.05) between a genus and a particular successional stage based on the indicator value analysis using the point-biserial correlation coefficient as the association index.
