# Supplementary material for: System Performance Corresponding to Bacterial Community Succession after a Disturbance in an Autotrophic Nitrogen Removal Bioreactor
Source: mSystems. 2020 Jul 21;5(4):e00398-20. doi: 10.1128/mSystems.00398-20 (PMC7566277; doi:10.1128/mSystems.00398-20)
Supplement: TABLE S3 [file mSystems.00398-20-st003.pdf]

**(a)**	**Richness**		**Shannon**		**Evenness**	
Successional stage	Mean	CV(%)	Mean	CV(%)	Mean	CV(%)
Early	306	19.06	4.79	5.12	0.58	4.93
Middle	357	17.21	5.46	6.03	0.65	4.27
Final	375	15.73	5.37	3.82	0.63	3.41
Whole	352	18.32	5.26	7.14	0.62	5.64

**(b)**	**Temperature**		**pH**		**O_2**	
Successional stage	Mean	CV(%)	Mean	CV(%)	Mean	CV(%)
Early	28.86	3.70	7.84	2.02	0.41	58.48
Middle	29.35	2.34	7.61	2.42	0.25	56.72
Final	29.93	0.17	7.52	1.12	0.13	26.51
Whole	29.35	3.07	7.66	2.64	0.24	85.94
