# Supplementary material for: System Performance Corresponding to Bacterial Community Succession after a Disturbance in an Autotrophic Nitrogen Removal Bioreactor
Source: mSystems. 2020 Jul 21;5(4):e00398-20. doi: 10.1128/mSystems.00398-20 (PMC7566277; doi:10.1128/mSystems.00398-20)
Supplement: TABLE S4 [file mSystems.00398-20-st004.pdf]

Successional stage	Mean	CV	MK tau[#]	(p-value)	Sen's slope[*]	(95% CI for slope)
Early	0.553	32%	**-0.471**	**<0.001**	**-0.026**	**(-0.035 ~ -0.005)**
Middle	0.453	31%	**0.692**	**<0.001**	**0.019**	**(0.014 ~ 0.025)**
Last	0.627	20%	-0.179	0.284	-0.003	(-0.009 ~ 0.004)

[#]A significant tau indicates an increasing (+) or decreasing (-) trend.

[*]The Sen's slope indicates the magnitude of the trend.
