# Supplementary material for: System Performance Corresponding to Bacterial Community Succession after a Disturbance in an Autotrophic Nitrogen Removal Bioreactor
Source: mSystems. 2020 Jul 21;5(4):e00398-20. doi: 10.1128/mSystems.00398-20 (PMC7566277; doi:10.1128/mSystems.00398-20)
Supplement: TABLE S5 [file mSystems.00398-20-st005.pdf]

ID	Genus	FAPROTAX# functional annotation
G1	*Nitrospira*	Aerobic nitrite oxidation and/or Aerobic ammonia oxidation
G2	*Candidatus* Jettenia	Anammox (*i.e.* anaerobic ammonia oxidation)
G3	*Nitrosomonas*	Aerobic ammonia oxidation and/or Ureolysis
G4	Groundwater metagenome	Unassigned (belonging to Planctomycetes anammox bacterium*)
G5	AKYH767 (uncultured)	Unassigned (belonging to Bacteroidetes bacterium*)
G6	SM1A02 (uncultured)	Unassigned (belonging to Planctomycetes anammox bacterium*)
G7	*Denitratisoma*	Nitrate denitrification and/or Aerobic chemoheterotrophy
G8	*Sideroxydans*	Dark iron oxidation
G9	OLB12 (uncultured)	Unassigned (belonging to Bacteroidetes bacterium*)
G10	*Bryobacter*	Aerobic chemoheterotrophy

#The FAPROTAX database, which affiliates taxa with metabolic functions (particularly nitrogen and carbon cycling) based on the literature on cultured representatives.

*According to the hierarchical taxonomic grouping.
